# Classical swine fever virus Shimen infection increases p53 signaling to promote cell cycle arrest in porcine alveolar macrophages

**DOI:** 10.18632/oncotarget.18997

**Published:** 2017-07-05

**Authors:** Pengbo Ning, Congxia Hu, Xuepeng Li, Yulu Zhou, Aoxue Hu, Ya Zhang, Lifang Gao, Cunmei Gong, Kangkang Guo, Xianghan Zhang, Yanming Zhang

**Affiliations:** ^1^ School of Life Science and Technology, Xidian University, Xi’an, Shaanxi, PR China; ^2^ Engineering Research Center of Molecular and Neuro Imaging Ministry of Education, Xi’an, Shaanxi, PR China; ^3^ College of Science, Northwest A&F University, Yangling, Shaanxi, PR China; ^4^ Bio-ID Center, School of Biomedical Engineering, Shanghai Jiao Tong University, Shanghai, PR China; ^5^ Shaanxi Provincial People’s Hospital, Xi’an, Shaanxi, PR China; ^6^ College of Veterinary Medicine, Northwest A&F University, Yangling, Shaanxi, PR China

**Keywords:** CSFV Shimen, p53 pathway, p21, cell cycle dysregulation, macrophages, Pathology Section

## Abstract

Classical swine fever virus (CSFV) replicates in macrophages and causes persistent infection. Despite its role in disastrous economic losses in swine industries, the molecular mechanisms underlying its pathogenesis are poorly understood. The virus evades the neutralizing immune response, subverting the immune system to ensure its own survival and persistence. Our genome-wide analysis of porcine alveolar macrophage transcriptional responses to CSFV Shimen infection using the Solexa/Illumina digital gene expression system revealed that p53 pathway components and cell cycle molecules were differentially regulated during infection compared to controls. Further, we investigated the molecular changes in macrophages infected with CSFV Shimen, focusing on the genes involved in the p53 pathway. CSFV Shimen infection led to phosphorylation and accumulation of p53 in a time-dependent manner. Furthermore, CSFV Shimen infection upregulated cyclin-dependent kinase inhibitor 1A (p21) mRNA and protein. In addition, CSFV Shimen infection induced cell cycle arrest at the G1 phase, as well as downregulation of cyclin E1 and cyclin-dependent kinase 2 (CDK2). The expression of genes in the p53 pathway did not change significantly after p53 knockdown by pifithrin-α during CSFV Shimen infection. Our data suggest that CSFV Shimen infection increases expression of host p53 and p21, and inhibits expression of cyclin E1 and CDK2, leading to cell cycle arrest at the G1 phase. CSFV may utilize this strategy to subvert the innate immune response and proliferate in host cells.

## INTRODUCTION

Macrophages play an important role in both innate and acquired immune responses [1]. Initially, macrophages are required to kill microorganisms such as viruses when they enter the body [2]. However, viruses are often able to survive through co-evolution with their hosts. Macrophages are often attacked during viral infection and become carriers of the virus, leading to chronic infection [3].

Classical swine fever (CSF), caused by classical swine fever virus (CSFV), is a highly contagious disease affecting swine and wild boar; it is listed as a notifiable disease by the World Animal Health Organization [4]. Acute CSF, caused by a virulent strain of CSFV, presents with a typical pathology, including hemorrhagic lymphadenitis and diffuse hemorrhage in the skin, kidneys, and other organs [5]. Over the past few decades, the epidemic of acute CSF has been effectively controlled by administration of the CSFV C strain vaccine and culling. However, the incidence of chronic and atypical CSF is increasing, which makes prevention and control difficult owing to the inability to distinguish this disease from other pig diseases [6]. A cellular microenvironment that facilitates virus replication is essential for the establishment of CSFV infection [7]. Although macrophages play a crucial role in protecting the host from viral infection, their protective activities are subverted by CSFV [8], which could cause persistent infection in pigs.

Despite progress in CSFV pathogenesis research, the key mechanism by which CSFV subverts the immune system is still poorly understood. The characteristics of the cellular microenvironment are likely to determine the outcome of the infection. To investigate the mechanism of CSFV pathogenesis, we performed digital gene expression (DGE) profiling to identify key genes that regulate signaling pathways in macrophages during infection. Furthermore, we investigated the effects of viral infection on the genes involved in transformation related protein (Trp53 or p53) signaling and cell cycle progression in macrophages.

## RESULTS

### p53 signaling pathway as the most enriched pathway in CSFV Shimen-infected macrophages

**Figure 1:**
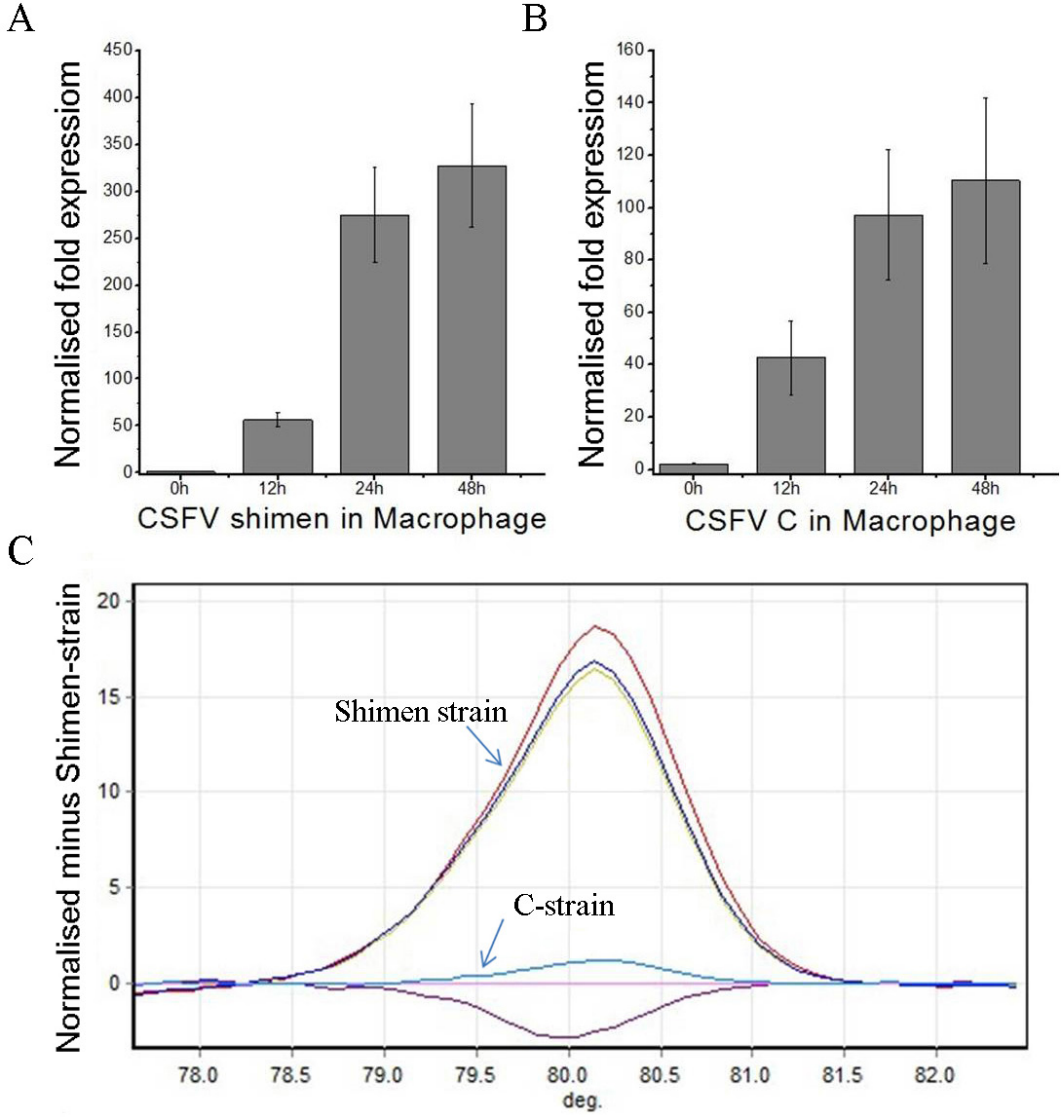
qPCR and high resolution melt analysis of CSFV Shimen and C strain proliferation in macrophages **A.** qPCR analysis of the proliferation of CSFV Shimenin macrophages. **B.** qPCR analysis of the proliferation of CSFV C strain in macrophages. **C.** High resolution melt analysis of the proliferation of CSFV Shimenand C strains in macrophages in the absence of cross-infection. The results are representative of three independent experiments, and data is expressed as mean ± SEM.

To determine the extent of proliferation of the CSFV Shimen and C strains after infection, we assessed total viral RNA content in infected porcine macrophages (cell line CRL-2843) by real-time polymerase chain reaction (qPCR) (Figure 1A-1B). Both strains underwent exponential growth in macrophages in the 48-h period after infection. Given that the CRL-2843 cells maintained their morphology 48 h after infection, we selected this time point for the DGE study. As the CSFV Shimen and C strains have similar gene sequences and encoded proteins, we performed high resolution melt analysis to definitively identify the virus in each sample. As shown in Figure 1C, the high resolution melt results were consistent with those of the qPCR assay and confirmed infection with either CSFV Shimenor C with no cross-contamination between the samples.

To under stand the global molecular changes caused by infection with CSFV Shimen and C strains, we used the DGE system to explore the transcriptomes of macrophages infected with the two strains and an uninfected control. We constructed cDNA libraries for uninfected and CSFV (Shimen and C)-infected macrophages and analyzed their characteristics by high-throughput Illumina sequencing (Supplementary Table 1). All tags were aligned to the reference sequences in the NCBI UniGene *Susscrofa* database, and gene products with high homology to *Susscrofa* were identified by BLAST (Supplementary Table 2) [9].

**Figure 2:**
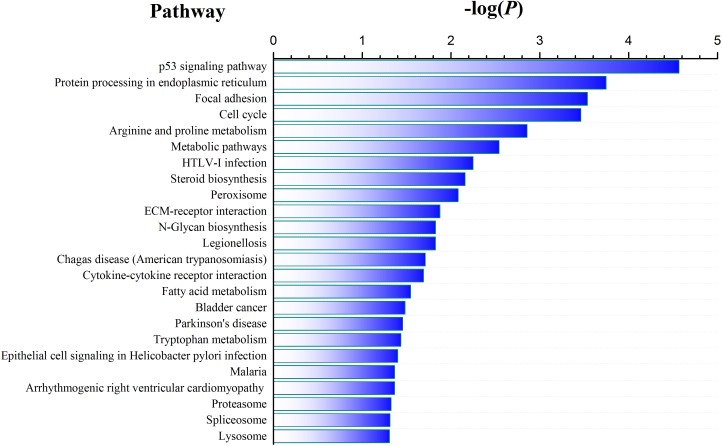
Pathway enrichment analysis in CSFV Shimen-infected versus control macrophage samples

To identify genes associated with CSFV Shimen infection, we analyzed genes that were differentially expressed among the 3 samples. As shown in Supplementary Figure 1, we observed differential expression of 1,676, 3,074, and 2,525 genes in the three comparisons (Shimen vs. control, C vs. control, and Shimen vs. C, respectively) (false discovery rate ≤ 0.001 and |log2Ratio| ≥ 1). We performed pathway analysis on the differentially expressed genes,using the Kyoto Encyclopedia of Genes and Genomes (KEGG) database,to gain insight into their functions. We found that the most significantly enriched pathway in the CSFV Shimen-infected sample compared to that in the control was the p53 signaling pathway (Figure 2). Interestingly, we did not detect a significant difference in the p53 signaling pathway when comparing CSFV C-infected and control samples (Supplementary Figure 2). The cell cycle pathway was also differentially regulated in the CSFV Shimen-infected and control samples. These results suggested that upregulation of the p53 signaling pathway by CSFV Shimen in macrophages may mediate their response to infection by modulating the cell cycle.

### CSFV Shimen promotes phosphorylation and expression of p53 in macrophages

**Figure 3:**
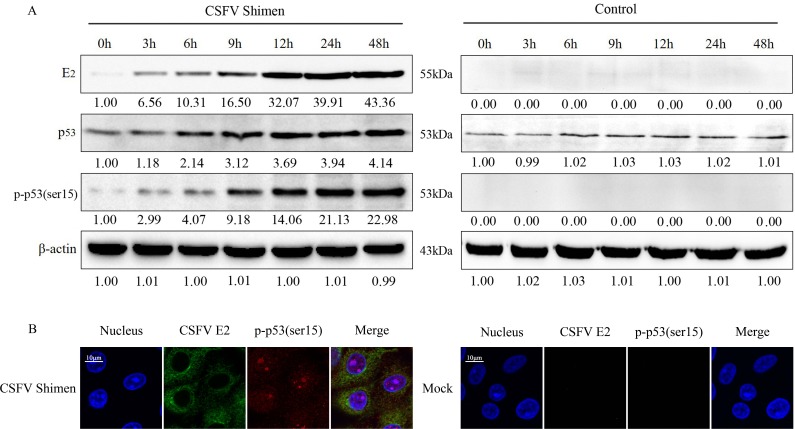
CSFV Shimen promoted the phosphorylation and expression of p53 in macrophages **A.** Macrophages were infected with CSFV Shimen for the indicated time. At the end of the infection, the expression of CSFV E2, p53, phospho-p53(ser15), and β-actin (loading control) were analyzed by immunoblotting with specific antibodies. The levels of the proteins at 0 h were considered 1-fold. Control group, mock-infected cells. **B.** Co-localization of CSFV E2 protein with phospho-p53(ser15) in macrophages infected with CSFV Shimen. After 24 h, macrophages were fixed with methanol and stained with phospho-p53(ser15) and CSFV E2 antibodies. Green, CSFV E2 fluorescein isothiocyanate-labeled antibody staining; red, phospho-p53(ser15) tetramethylrhodamineisothiocyanate-labeled antibody staining. The position of the nucleus is indicated by DAPI staining in blue. Merged images show significantly enhanced fluorescence representing phospho-p53(ser15) in macrophages corresponding to time post-infection with CSFV Shimen. The images are representative of 3 experiments with similar results. Bar = 10 μm, except for the enlarged figures.

We next examined whether CSFV Shimen infection influenced p53 expression in macrophages. We compared protein expression in both CSFV Shimen- and mock-infected macrophages. We observed a marked time-dependent increase in CSFV Shimen E2 protein expression in whole cell lysates of CSFV Shimen-infected cells (Figure 3A), which was absent in mock-infected cells, consistent with the qPCR analysis (Figure 1). Next, we investigated the levels and activity of p53 in infected macrophages. We found that Shimen infection promoted the accumulation of total cellular p53 3 h post-inoculation, and the levels remained elevated up to 12 h post-inoculation. Importantly, an increase in the level of phosphorylation of p53 at serine 15was observed in CSFV Shimen-infected macrophages at 0, 3, 6, 12, 24, or 48 hpost-infection (Figure 3A). We did not detect phospho-p53(Ser15) in the negative control at any of the time points tested (Figure 3A).

To assess the relationship between activated p53 and CSFV Shimen E2 protein, we stained macrophages at various time points post-infection with fluorescently labeled antibodies against CSFV Shimen E2 and phospho-p53(Ser15) and tracked their cellular localization by confocal microscopy. We observed co-localization of CSFV Shimen E2 protein and phospho-p53(Ser15), and the expression of both molecules significantlyincreased over the course of CSFV Shimen infection (Figure 3B). Our results suggested that CSFV Shimen infection activated the p53 signaling pathway.

### CSFV Shimen infection induces p21 expression

**Figure 4:**
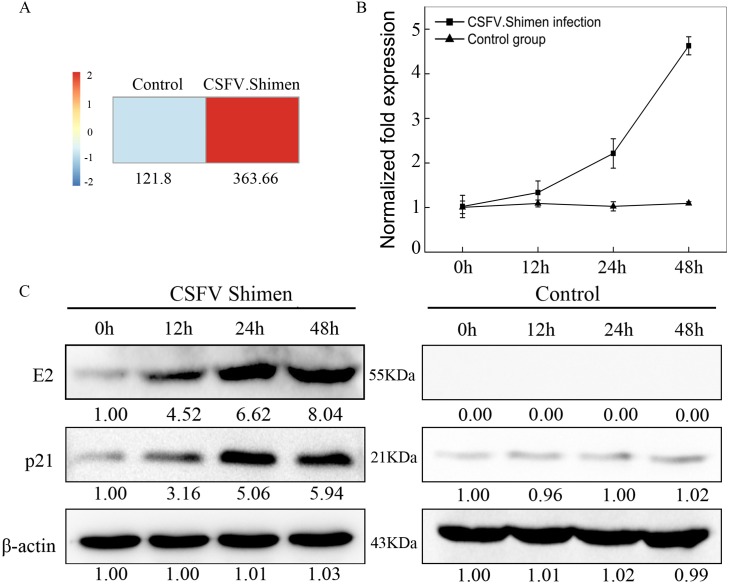
CSFV Shimen infection increased the expression of p21 mRNA and protein in macrophages **A.** DGE analysis of macrophages revealed that p21 correlated with CSFV Shimen infection when compared to CSFV C infection and negative controls (mock-infected cells). **B.** Analysis of p21 mRNA levels by qPCR on macrophages infected with CSFV Shimen and controls. β-actin was probed as the loading control. Control group, mock-infected cells. **C.** Effects of CSFV Shimen infection on p21 protein expression. Macrophages were infected with CSFV Shimen for 0, 12, 24, and 48 h. At the end of the infection, the expression of CSFV E2, p21, and β-actin (loading control) were analyzed by immunoblotting with specific antibodies. The levels of indicated proteins at 0 h were considered 1-fold. Control group, mock-infected cells.

Given that CSFV Shimen infection led to p53 accumulation and phospho-p53(Ser15) activation, we next examined which downstream molecules in the p53 signaling pathway were upregulated by infection. The DGE data revealed 3-fold higher expression of cyclin-dependent kinase inhibitor 1A (Cdkn1a or p21) in CSFV Shimen-infected macrophages than in controls 48 h post-infection (Figure 4A), suggesting increased p21 biosynthesis in virus-infected macrophages compared with that in controls. Increased transcription of p21, a downstream target of p53 [10], likely follows activation of p53 during CSFV Shimen infection. We analyzed the expression of p21 mRNA and protein in macrophages after infection with CSFV Shimen. As shown in Figure 4B, p21 transcript levels were significantly higher in CSFV Shimen-infected cells than in controls (up to 4.63 ± 0.20-fold). We also determined p21 protein expression levels in macrophages after infection with CSFV Shimen. Importantly, p21 accumulated in virus-infected macrophages (Figure 4C). We did not observe significant changes in p21 accumulation in the control cells.

### CSFV Shimen decreases cyclin E and CDK2 expression, resulting in cell cycle dysregulation

**Figure 5:**
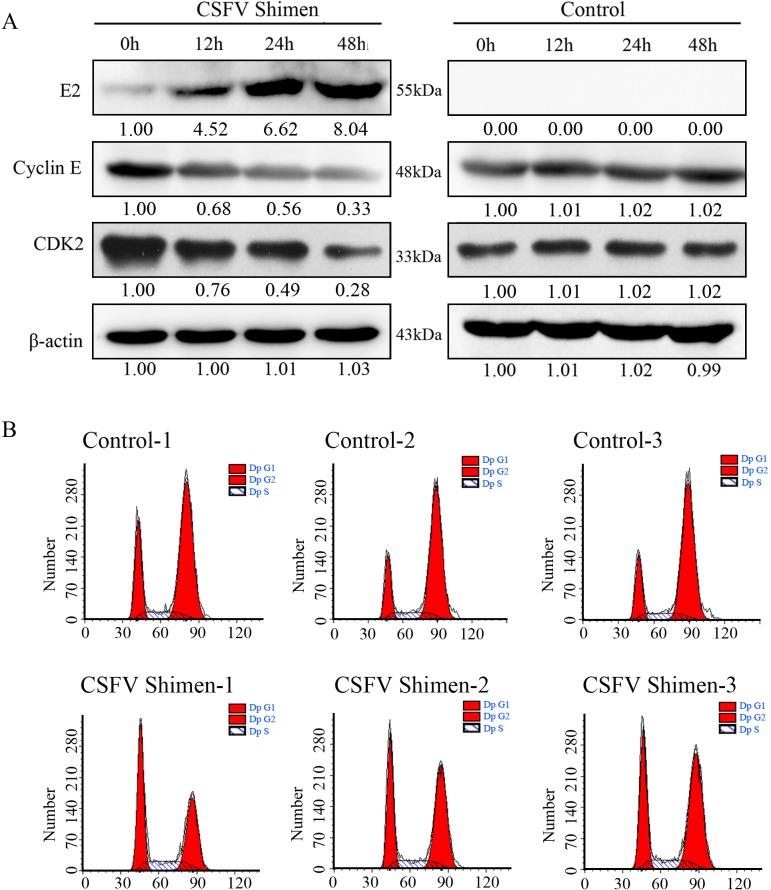
CSFV Shimen decreased cyclin E and CDK2 expression, which promoted cell cycle arrest **A.** Macrophages were infected with CSFV Shimen for 0, 12, 24, and 48 h. At the end of the infection, the expression of CSFV E2, cyclin E, CDK2, and β-actin (loading control) were analyzed by immunoblotting with specific antibodies. The levels of the proteins at 0 h were considered 1-fold. Control group, mock-infected cells. **B.** G1 arrest induced by the infection of CSFV Shimen in macrophages. Cell cycle status was determined by flow cytometry, as described in Materials and Methods. The results are representative of three independent experiments.

The cell cycle pathway is tightly regulated by a p53 transcription-dependent mechanism. As the DGE pathway analysis showed that CSFV Shimen infection activated the cell cycle pathway of macrophages (Figure 2), we wanted to investigate whetherdownstream cell cycle molecules were suppressed in infected cells. We found that the increase in E2 protein correlated with a decrease in cyclin E levels, as measured by western blot, in a time-dependent manner (Figure 5A). The expression of cyclin E was not altered in the control cells. In addition, we observed a dramatic reduction in CDK2 expression in CSFV Shimen-infected cells (Figure 5A). The G1-to-S phase cell cycle transition is controlled by specific interactions between cyclin E and CDK2. To assess the effects of CSFV Shimen infection on this transition, we synchronized cells in pseudo-G0 phase by serum withdrawal [11] before viral infection. Macrophages were collected at 48 h post-infection, with uninfected macrophages serving as controls. Flow cytometric analysis revealed that CSFV Shimen infection suppressed the cell cycle in macrophages and retained cells in the G1 phase (Figure 5B).

### CSFV Shimen induces cell cycle arrest via p53

**Figure 6:**
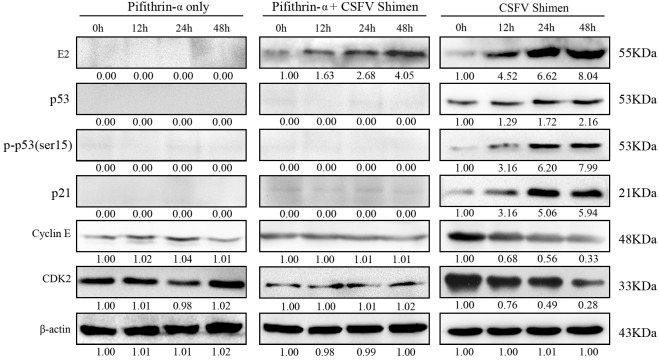
Activation of p53 in CSFV Shimen-infected macrophages is required for cell cycle arrest To determine the effect of p53 in CSFV Shimen-induced cell cycle arrest, p53 was depleted with the inhibitor PFT-α in CSFV Shimen-infected and mock-infected macrophages. A significant reduction in p53 protein synthesis was detected in the presence of PFT-α (Lane 1 in the left panel and the middle panel). In the middle panel, pre-treatment of macrophages with PFT-α blocked CSFV Shimen-induced phosphorylation of p53 and blocked the upregulation of p21. The levels of cyclin E and CDK2 were not reduced and no significant changes were observed in PFT-α-treated cells (middle panel), compared to CSFV Shimen-infected cells without PFT-a treatment (the right panel).

Our results suggested that CSFV Shimen infection induced phosphorylation of p53(Ser15) and expression of p21. We treated cells with pifithrin-α (PFT-α), a specific inhibitor that blocks transcription of p53-responsive genes, to investigate the role of p53 in CSFV Shimen-induced cell cycle arrest [12]. We examined the levels of p53 and phospho-p53(Ser15) in macrophages after PFT-α treatment. We found, by western blot, that treatment diminished p53 protein expression (Figure 6, left panel). We also observed that pre-treatment of macrophages with PFT-α blocked the CSFV Shimen-induced phosphorylation of p53 and expression of p21 (Figure 6, middle panel). We did not detect alterations to the levels of cyclin E and CDK2 in PFT-α treated cells (Figure 6, middle panel) compared to the levels in infected cells without PFT-α treatment (Figure 6, right panel).

Taken together, our findings demonstrated that CSFV Shimen increased p53 signaling, leading to upregulation of p21 and concomitant downregulation of cyclin E1 and CDK2 in a time-dependent manner, resulting in cell cycle arrest at the G1 phase.

## DISCUSSION

CSF is one of the most severe diseases that affect pigs worldwide, with massive economic consequences [13]. Macrophages are the immune cell type primarily responsible for the phagocytosis of pathogens and activation of the immune system. However, macrophages are also target cells for CSFV, and the migration and diffusion of macrophages *in vivo* is an important mechanism of infection for CSFV [14]. Thus, identification of the mechanisms by which viruses subvert the immune system could provide insight into virus infection strategies.

We investigated the mechanism of CSFV infection of macrophages by DGE. We compared macrophages infected with the CSFV Shimen strain to cells infected with the CSFV C strain and uninfected controls to elucidate the mechanism of persistent CSFV infection. CSFV Shimen is a virulent strain, which causes the typical clinical symptoms of swine fever [15]. CSFV C is an attenuated strain that does not cause pathological symptoms [16]. Comparison of the differences in gene regulation between CSFV Shimen infection and C strain or mock infection by DGE analysis facilitates an understanding of the pathogenesis of the infectious strain.

In this study, macrophages with CSFV Shimen, CSFV C, or mock infection were analyzed after 48 h by DGE. We identified differential responses to CSFV Shimen in the host macrophages compared to that in the controls.Interestingly, pathway analysis suggested that CSFV Shimen triggered the accumulation and activation of p53, which promoted cell cycle arrest in macrophages.

The p53 signaling pathway plays an important role in DNA damage repair [17];when DNA is damaged or cell proliferation is abnormal, p53 is activated, resulting in cell cycle arrest and DNA repair. Recent studies have demonstrated that when a cell is infected by a virus, p53 is involved in determining the fate of the cell [18]. Viruses activate or interfere with the p53 signaling pathway at different levels to achieve proliferation. Hepatitis B virus interferes with the function of p53 by blocking its nuclear localization and binding with DNA [19]. The core protein of hepatitis C virus activates p53 during the infection process [20]. Avian reovirus p17 protein regulates cell cycle and autophagy by activating the p53/PTEN pathway [21]. Infectious bursal disease virus infection increases the activity of chicken p53, which is targeted by gga-miR-2127 [22]. A recent study indicates CAV1-mediated endocytosis is advantageous for productive CSFV Shimen infection in macrophages [8]. As a principal component of caveolae membranes in vivo, caveolin-1 expression negatively regulates cell cycle progression by inducing G0/G1 arrest via a p53/p21WAF1/Cip1-dependent mechanism [23]. The reported researches are beneficial to a deep-going understanding to the activation of p53 signaling pathway by CSFV Shimen.

Our observations were consistent with those in a previous report [24], which suggested that activation of p53 causes accumulation of p21, a cyclin-dependent kinase inhibitor, and eventual induction of downstream target genes. Both p21 and p53 cause cell cycle arrest at the G1 checkpoint, which promotes DNA repair by allowing sufficient time for damaged DNA to be repaired before it is passed to daughter cells [25, 26]. The mechanisms used by viruses to manipulate p53 and induce p21 expression are complex and diverse. For example, hepatitis B virus X protein can circumvent the need for p53 and induce transcription of the gene for p21 [27], while hepatitis C virus NS2 protein cooperates with p53 to inhibit DNA damage in cells but cannot induce the expression of p21 [28].

**Figure 7:**
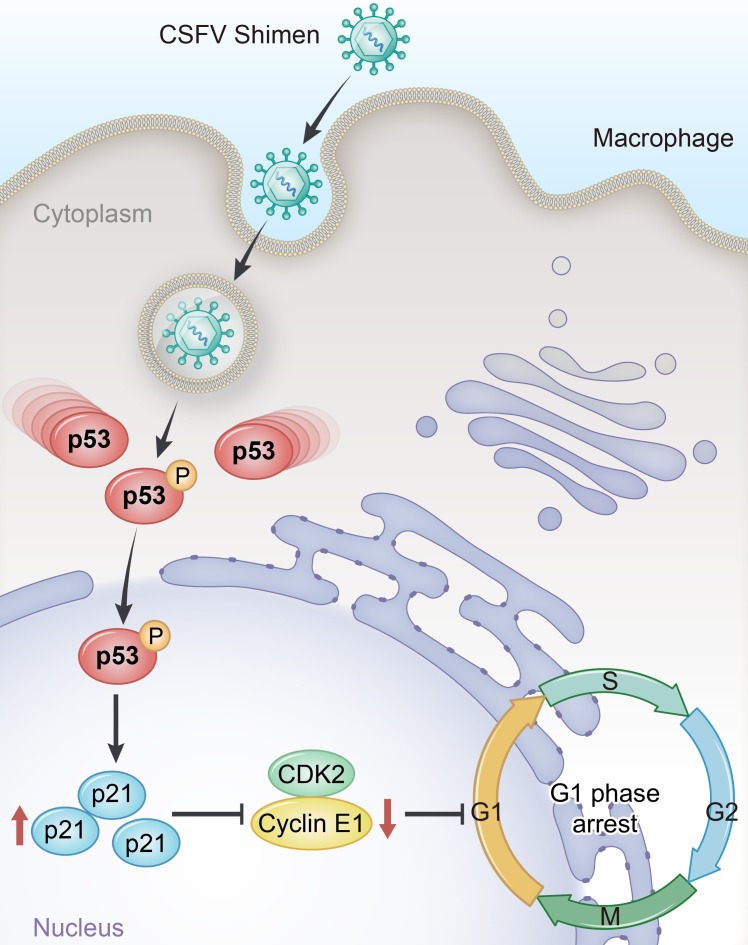
Proposed mechanism for upregulated p53 signaling to promote cell cycle arrest in porcine alveolar macrophages induced by CSFV Shimen

The main target of p21 is the cyclin E-CDK2 complex, in addition to the cyclin D-CDK4/6 complex [29]. Cyclin E participates in the regulation of cell cycle progression [30]. If the same signaling event causes the synthesis of cyclin E, the GI-to-S phase transition is initiated [31]. The Cyclin E-CDK2 complex is inhibited by p21 during the progression from G1 to S phase [32]. Cells regulate the p53 pathway, which controls the cell cycle, in order to maintain their stability and modulate their differentiation [33]. Researchers are investigating the infection strategies that facilitate viral proliferation by manipulating the cell cycle. However, research into the role of p53 signaling pathway in CSFV infection is still in its nascent stage. Pathway analysis of our DGE data indicated CSFV Shimen infection induced significant differences in the p53 signaling pathway and cell cycle response compared to the mock- and C strain-infection. Bioinformatics analysis highlighted the importance of the p53 signaling pathway in CSFV Shimen infection. We found that CSFV Shimeninfection in macrophages promoted phosphorylation of p53(Ser15) and increased the expression of p21. Thus, infection with CSFV Shimen activated p53, resulting in upregulation of p21, inhibition of cyclin E-CDK2, and, ultimately, cell cycle arrest, which could facilitate persistent infection (Figure 7).

In conclusion, the present DGE study showed that the p53 signaling pathway was significantly altered in response to CSFV Shimen infection in macrophages. We observed an increase in p53 and p21 levels in CSFV Shimen-infected macrophages, an arrest of the cell cycle, and cyclin E and CDK2 downregulation. Furthermore, we showed that the effects of both p53 and genes involved in the p53 pathway could be inhibited by treatment with PFT-a. We concluded that CSFV Shimen infection may be responsible for enhanced activation of the p53 pathway and is involved in G1 arrest in response to p21 activation. The p53 signaling pathway appears to be the most important regulatory pathway for CSFV Shimen in macrophages, and most likely represents the key mechanism used by the virus to escape host immune clearance and establish persistent infection.

## MATERIALS AND METHODS

### Culture of macrophages, CSFV infection, and RNA isolation

The porcine alveolar macrophage cell line 3D4/21 (CRL-2843) was obtained from the American Type Culture Collection (Manassas, VA, USA) [8]. The CSFV Shimen and C strains were obtained from the National Control Institute of Veterinary Bio products and Pharmaceuticals (Beijing, China). Macrophages were cultured in 25-cm2 tissue culture flasks at a density of 2 × 107 cells per flask, and the CSFV Shimen and C strains were added to the cultures at a multiplicity of infection of 5 (TCID50) when macrophages were 70%-80% confluent [8]. After 1 h of incubation at 37°C in 5% CO2, the medium was aspirated, fresh medium containing 2% fetal calf serum was added, and the cultures were incubated for 48 h in 5% CO2. High resolution melt analysis was conducted to confirm infection with CSFV Shimen and C strains, and qPCR was carried out to detect the proliferation of CSFV [34]. Total RNA was isolated from CSFV-infected macrophages and control samples at 48 h post-infection, using TRIzol® reagent (Invitrogen, Carlsbad, CA, USA), according to the manufacturer’s protocol. RNA yields were determined by absorbance of samples at 260 nm using a NanoDrop (ND-2000; NanoDrop Technologies, Wilmington, DE, USA). An Agilent 2100 Bioanalyzer (Agilent Technologies, Palo Alto, CA, USA) was used to evaluate RNA integrity. CSFV Shimen-infected, CSFV C-infected, and control macrophage samples were separately subjected to DGE profiling based on Solexa sequencing.

### Solexa sequencing and DGE tag profiling analysis

We created cDNA librariesby our previous report [9], using the Illumina Gene Expression Sample Prep kit (Illumina, San Diego, CA, USA). Raw reads were filtered through the Illumina pipeline to obtain clean tags, which were normalized to the number of transcripts per million clean tags [9, 35]. The following formula was used to demonstrate the probability that one gene was equally expressed in two samples [36]:

$$p(y|x)={\left(\frac{N_{2}}{N_{1}}\right)}^{y}\frac{(x+y)!}{x!y!{\left(1+\frac{N_{2}}{N_{1}}\right)}^{\left(x+y+1\right)}}$$

where*x* and *y* indicate the clean tags mapping to the gene. *N1* and *N2* represent the total number of clean tags in two compared libraries. The *p*-value corresponds to differential gene expression. The false discovery rate determines the threshold of the *p*-value in multiple tests and analyses [37]. In this study, the statistical significance of differences in gene expression were determined using the threshold false discovery rate ≤ 0.001 and the absolute value of log2 ratio ≥ 0.

Pathway enrichment analyses identify significantly enriched metabolic pathways or signal transduction pathways from differentially expressed gene data by the following formula [38, 39]:

$$p=1-\sum_{i=0}^{m-1}\frac{\left(\begin{array}{c} M\\ i \end{array}\right)\left(\begin{array}{c} N-M\\ n-i \end{array}\right)}{\left(\begin{array}{c} N\\ n \end{array}\right)}$$

where*N* is the total number of genes with KEGG functional annotations, and *n* is the number of differentially expressed genes in *N*. *M* is the number of the genes with specific KEGG annotations, and *m* is the number of differentially expressed genes in *M*.

### Western blot analysis

Protein extraction and western blot were performed as previously described [40, 41]. Protein concentration was determined using a BCA Protein Assay Kit (Cowin Biotech, Beijing, China). Equivalent amounts of proteins were subjected to 12% sodium dodecyl sulfate-polyacrylamide gel electrophoresis and transferred to polyvinylidenedifluoride membranes (Millipore, Atlanta, GA, USA). The membranes were blocked for 2 h at 25°C± 2°C in Tris-buffered saline containing 0.05% Tween® 20 and 5% nonfat milk, and incubated with specific primary antibodies raised against p53 (1C12; Cell Signaling Technology, Beverly, MA, USA), phospho-p53(Ser15) (Cell Signaling Technology), p21Waf1/Cip1 (12D1; Cell Signaling Technology), cyclin E1 (HE12; Cell Signaling Technology), CDK2 (M2; Santa CruzBiotechnology, Santa Cruz, CA, USA), and β-actin (Tianjin Sungene Biotech, Tianjin, China) at 4°C overnight, and with the corresponding secondary antibody conjugated to horseradish peroxidase at appropriate dilutions for 1 h at 37°C. Images were captured using a GeneGnome XRQ chemiluminescence detector (Syngene, Cambridge, UK); the densities of the protein bands were normalized to the β-actin signal and quantified using GeneSys software (VilberLourmat, France). The abundance of the proteins of interest in the various treatments was expressed relative to the abundance under control conditions.

### Confocal immunofluorescence microscopy

CSFV-infected cells were washed in phosphate-buffered saline (PBS) and fixed with methanol/acetone (1:1) for 20 min at 25°C ± 2°C and permeabilized with 1% Triton X-100 in PBS for 10 min. After three washes in PBS, samples were incubated with mouse anti-E2 antibody (MssBio, Guangzhou, China) and rabbit anti-phospho-p53(Ser15) (Cell Signaling Technology) for 1 h at 25°C ± 2°C, followed by staining with donkey anti-rabbit IgG conjugated to Alexa Fluor® 594 and donkey anti-mouse IgG conjugated to Alexa Fluor® 488 (YEASEN, Shanghai, China) at a 1:200 dilution for 1 h at 25°C ± 2°C. After incubation with 4′, 6-diamidino-2-phenylindole (DAPI), the samples were observed using a laser-scanning confocal microscope (LSM 510 META; Carl Zeiss, Jena, Germany).

### Flow cytometry

After infection or mock infection, cells were harvested and fixed in 70% ethanol for 30 min on ice. Cells were washed, pelleted in PBS, and resuspended in propidium iodide solution (50 mg/mL) containing 0.1 mg/mL RNase for 40 min at 37°C. Flow cytometry was performed to examine cell cycle status, using a Coulter® EPICS® XL/MCL Flow Cytometer (Beckman Coulter, Hialeah, FL, USA). We acquired 10,000 events for each sample. Data were analyzed using Expo32 ADC software (Beckman Coulter, Miami, FL, USA).

### qPCR analysis

We performed qPCR on a Bio-Rad iQ™5 system (Bio-Rad, California, USA) with SYBR® Premix Ex Taq™ II (TaKaRa, Dalian, China), on the same RNA samples used for the DGE experiments. We synthesized cDNA, using the Transcriptor First Strand cDNA Synthesis Kit (TaKaRa, Dalian, China), according to the manufacturer’s instruction. Each cDNA sample was analyzed in triplicate, and the average threshold cycle (Ct) was calculated per sample. The 2-ΔΔCt method [42] was applied to calculate the relative expression levels among CSFV-infected and control samples.

### Statistical analysis

Statistical analyses were conducted by one-way or two-way analysis of variance, using the Statistical Package for the Social Sciences (SPSS) 16.0 (SPSS, Chicago, IL, USA), and differences with a *p*-value < 0.05 were considered statistically significant. Data are shown as the mean ± standard error of mean (SEM) of three independent experiments.

## SUPPLEMENTARY MATERIALS FIGURES AND TABLES





## Abbreviations

CSFV: classical swine fever virus
p53: transformation related protein 53
p21: cyclin-dependent kinase inhibitor 1A
CDK2: cyclin-dependent kinase 2
CSF: classical swine fever
DGE: digital gene expression
qPCR: real-time polymerase chain reaction
phospho-p53(ser15): phosphorylation of p53 at serine 15
KEGG: Kyoto Encyclopedia of Genes and Genomes
PFT-α: pifithrin-α
PBS: phosphate-buffered saline
DAPI: 4′, 6-diamidino-2-phenylindole
Ct: threshold cycle
SPSS: Statistical Package for the Social Sciences
SEM: standard error of the mean
