# Supplementary material for: Classical swine fever virus Shimen infection increases p53 signaling to promote cell cycle arrest in porcine alveolar macrophages
Source: Oncotarget. 2017 Jul 5;8(34):55938–49. doi: 10.18632/oncotarget.18997 (PMC5593535; doi:10.18632/oncotarget.18997)
Supplement: Supplementary file 1 [file oncotarget-08-55938-s001.pdf]

Classical swine fever virus Shimen infection increases p53 signaling to promote cell cycle arrest in porcine alveolar macrophages

SUPPLEMENTARY MATERIALS

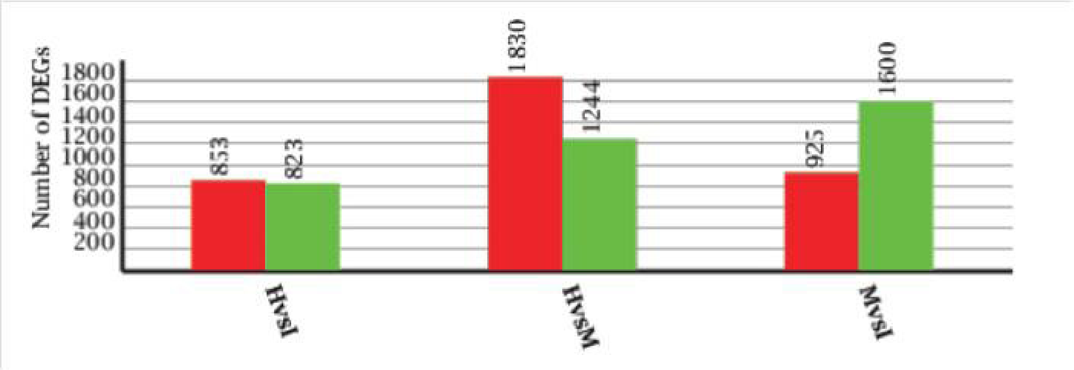

Supplementary Figure 1: Differentially expressed genes identified by pairwise comparisons among Control, CSFV-C, and CSFV-Shimen groups

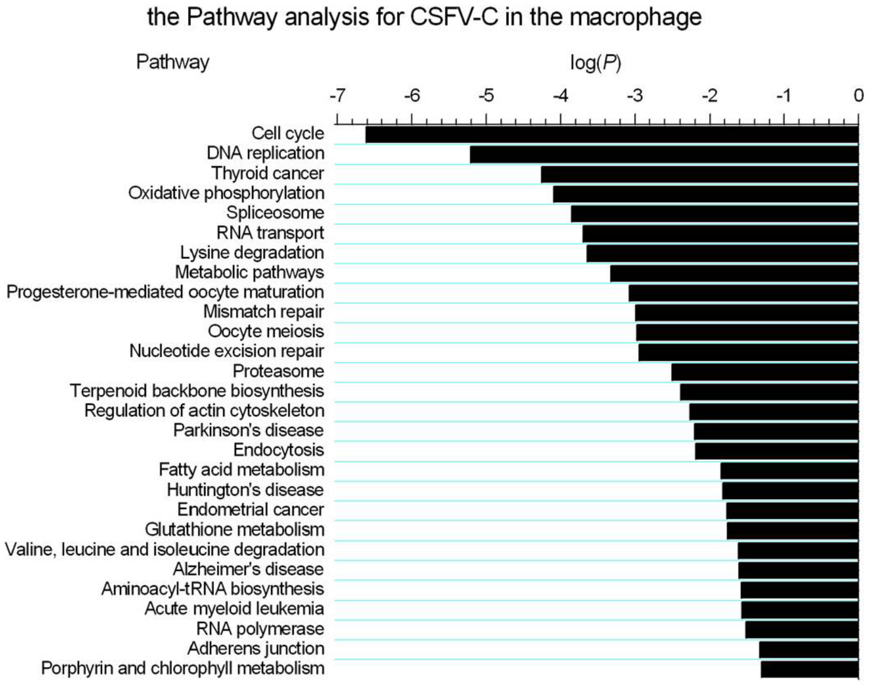

Supplementary Figure 2: Pathway enrichment analysis for genes in CSFV C-infected macrophages vs. mock-infected macrophages

Supplementary Table 1: Summary statistics of tags in CSFV presence and control samples.

Summary		Control	CSFV Shim	CSFV C
Raw Data	Total	5000000	5000000	4888392
Raw Data	Distinct Tag	296819	306634	484401
Clean Tag	Total number	4811227	4809387	4423399
Clean Tag	Distinct Tag number	112250	119252	157818
All Tag Mapping to Gene	Total number	3860602	3781218	3401590
All Tag Mapping to Gene	Total % of clean tag	80.24%	78.62%	76.90%
All Tag Mapping to Gene	Distinct Tag number	62543	64854	76966
All Tag Mapping to Gene	Distinct Tag % of clean tag	55.72%	54.38%	48.77%
Unambiguous Tag Mapping to Gene	Total number	3465087	3373271	3033627
Unambiguous Tag Mapping to Gene	Total % of clean tag	72.02%	70.14%	68.58%
Unambiguous Tag Mapping to Gene	Distinct Tag number	56103	58688	68141
Unambiguous Tag Mapping to Gene	Distinct Tag % of clean tag	49.98%	49.21%	43.18%
All Tag-mapped Genes	number	19454	20052	21265
All Tag-mapped Genes	% of ref genes	41.90%	43.18%	45.80%
Unambiguous Tag-mapped Genes	number	17665	18247	19532
Unambiguous Tag-mapped Genes	% of ref genes	38.04%	39.30%	42.06%
Mapping to Genome	Total number	561207	613433	619939
Mapping to Genome	Total % of clean tag	11.66%	12.75%	14.01%
Mapping to Genome	Distinct Tag number	31286	34935	51242
Mapping to Genome	Distinct Tag % of clean tag	27.87%	29.30%	32.47%
Unknown Tag	Total number	389418	414736	401870
Unknown Tag	Total % of clean tag	8.09%	8.62%	9.09%
Unknown Tag	Distinct Tag number	18421	19463	29610
Unknown Tag	Distinct Tag % of clean tag	16.41%	16.32%	18.76%

For Supplementary Table 2 see in Supplementary Files
